# Analysis of Morbidity and Mortality Due to Yellow Fever in Brazil

**DOI:** 10.3390/v17030443

**Published:** 2025-03-19

**Authors:** Luisa Sousa Machado, Antonio Francisco Marinho Sobrinho, Andrielly Gomes De Jesus, Juarez Antônio Simões Quaresma, Helierson Gomes

**Affiliations:** 1 Federal University of Northern Tocantins (UFNT), Araguaína 77826-612, TO, Brazil; 2 UFT Tropical Diseases Hospital (HDT/UFT), Federal University of Northern Tocantins (UFNT), Araguaína 77803-120, TO, Brazil; 3 Evandro Chagas Institute, Ananindeua 67000-000, PA, Brazil; juarez.quaresma@gmail.com; 4 State University of Pará, Belém 66050-000, PA, Brazil; 5 Federal University of Pará, Belém 66075-110, PA, Brazil; 6 Federal University of São Paulo, São Paulo 01246-904, SP, Brazil

**Keywords:** yellow fever, epidemiology, vaccination, environmental health, socioeconomic factors

## Abstract

Introduction: Yellow fever (YF) is a viral hemorrhagic fever transmitted by mosquitoes, characterized by a high mortality due to kidney and liver failure, massive coagulation disorders, and hemorrhages. With no specific treatment, prevention through vaccination and vector control is essential. This study investigates the epidemiology of YF in Brazil from 2011 to 2020, focusing on its trends and distribution across the territory. Methods: This ecological time-series study analyzed confirmed YF cases in Brazil’s 27 federative units between 2011 and 2020. Data were sourced from DATASUS, IBGE, and IPEA. Incidence rates per 100,000 inhabitants were calculated, and various sociodemographic and health indicators were analyzed. Prais–Winsten autoregressive models assessed the trends, while a spatial analysis identified the risk areas using global and local Moran’s I statistics. The data were processed using Stata and GeoDa^®^ software, version 1.12. Results: YF cases were concentrated in the Amazon and Atlantic Forest biomes. The majority of the cases occurred in males (83.3%), non-white individuals (94.3%), and rural workers. Pará showed an increasing trend in incidence. A higher vaccination coverage correlated with a lower YF incidence, though endemic areas with good vaccination coverage still exhibited high rates. Health and socioeconomic indicators were inversely related to incidence, highlighting disparities in regional development. Conclusion: Effective YF control requires multidisciplinary strategies, including expanded vaccination coverage, intensified vector control, and active surveillance. Research should focus on developing better vaccines, monitoring immunity, and improving the global response coordination.

## 1. Introduction

Yellow fever (YF) is a mosquito-borne viral hemorrhagic fever known for its high fatality rate. Its clinical manifestations include liver dysfunction, renal failure, coagulopathy, and shock. Understanding the influence of climate change and deforestation on urban cases is crucial, as is comparing the sylvatic and urban forms of the disease. Epidemiological surveillance is essential for controlling yellow fever, as global epidemiological data provide a comprehensive view of the spread of the disease. In addition, exploring the virology, pathogenesis, and histopathology of YF are fundamental to understanding the clinical presentation and natural history of the disease [1].

The yellow fever virus (YFV) is an arbovirus belonging to the genus *Orthoflavivirus* within the family *Flaviviridae*. [2]. Structurally, it is composed of a protein capsid, a positive-sense single-stranded RNA genome, and a lipid envelope. The capsid protects the genetic material of the virus, while the lipid envelope, derived from the host cell during budding, contains glycoproteins essential for viral infection, including the envelope protein (E) and the membrane protein (prM/M) [3,4,5]. The E protein is particularly important for the entry of the virus into host cells and in the induction of the immune response [5].

The YFV’s life cycle begins with the adsorption of the virus to the host cell, mediated by the E protein, which binds to specific receptors on the cell surface. After binding, the virus is internalized by endocytosis. Inside the endosome, acidification promotes the fusion of the viral envelope with the endosomal membrane, releasing the viral RNA into the host cell cytoplasm. In the cytoplasm, the viral RNA is translated into a polyprotein that is processed into individual proteins, including the nonstructural proteins NS3 and NS5, which are crucial for viral RNA replication [3,6].

The translation of yellow fever virus (YFV) proteins occurs in the cytoplasm of the host cell, where its single-stranded positive-sense RNA (ssRNA+) genome directly functions as messenger RNA (mRNA). This genome is translated into a single viral polyprotein, which is cleaved by viral and host proteases to generate structural and non-structural proteins. The structural proteins include C (capsid), which protects the genetic material, prM/M (pre-membrane/membrane), involved in viral envelope maturation, and E (envelope), essential for viral entry into the host cell [7,8].

The non-structural proteins (NS) play crucial roles in RNA replication and immune evasion. NS1 contributes to viral replication and modulates the host immune response, while NS2A and NS2B assist in assembling the replication complex. NS3 functions as a protease and helicase, facilitating polyprotein cleavage, whereas NS4A and NS4B reorganize the intracellular membranes to optimize viral replication. NS5, the largest viral protein, acts as an RNA-dependent RNA polymerase, essential for viral genome synthesis. This coordinated process enables the assembly of new virions, ensuring the YFV propagation and the establishment of infection in the host [7,8].

The RNA is replicated in replication complexes associated with the membranes of the endoplasmic reticulum. New viral particles are assembled in the endoplasmic reticulum and the Golgi complex and finally transported to the plasma membrane, where they are released by exocytosis—which consists of viral particles acquiring their envelope by fusing with intracellular membrane compartments before being secreted from the host cell [5,9].

The pathogenesis of yellow fever involves significant damage to host tissues and organs. The liver is the main target organ, where the virus causes hepatocellular necrosis, especially affecting the middle zone of the hepatic lobule. This necrosis is characterized by the presence of Councilman bodies, which are apoptotic hepatocytes [3,10]. Renal lesions include eosinophilic degeneration and fatty changes in the tubular epithelium, while myocardial damage is characterized by cellular degeneration and fatty changes [9,11]. Myocardial lesions consist of cellular degeneration and fatty changes in the myocardium. In the central nervous system, although rare, they may include inflammation and neuronal degeneration [3,9,12]. These lesions are responsible for the severe clinical manifestations of the disease, such as liver and kidney failure, and cardiovascular dysfunctions [10]. The bleeding diathesis, resulting from liver dysfunction and disseminated intravascular coagulation, contributes significantly to the high mortality of the disease [3,10].

During infection, the innate immune response is activated by the production of pro-inflammatory cytokines and the activation of NK cells. However, the YFV has mechanisms to inhibit this response, including the modulation of type I interferons by the NS5 protein and interference with the JAK-STAT pathway. The NS1 protein can also be secreted, where it acts as a soluble antigen that interferes with the complement response [9]. The adaptive immune response involves the activation of T and B cells, with the production of neutralizing antibodies against the YFV E protein. Immunity acquired after an infection or vaccination is generally long-lasting and protects against reinfection [9,10,11].

The progression of yellow fever can be divided into several stages. The incubation period lasts 3 to 6 days after infection, during which the patient is usually asymptomatic. The infectious period is characterized by a high fever, malaise, headache, photophobia, myalgia, anorexia, nausea, and vomiting, lasting about 3 to 4 days. This is followed by the remission period, where the fever and initial symptoms subside for 24 to 48 h, making the patient feel better temporarily. However, the intoxication period may follow, marked by the return of fever, jaundice, oliguria, hemorrhages, shock, and multiple organ failure, being critical and potentially fatal if not treated appropriately [3,11].

The diagnosis of yellow fever is essential for the appropriate management of the disease. Molecular methods, such as RT-qPCR, are the gold standard for the detection of viral RNA in blood or serum during the viremic phase, providing a rapid and accurate confirmation of infection [11]. In addition, serological tests such as ELISA are used to detect specific IgM and IgG antibodies against YFV, with the presence of IgM in a single serum sample being sufficient for a presumptive diagnosis [11,12]. Viral isolation, although less common due to the availability of molecular methods, can be performed through the inoculation of cell cultures or tissues [12]. Viral neutralization studies are also used to confirm the presence of specific neutralizing antibodies [11].

The treatment of yellow fever is predominantly supportive care. There are no specific antiviral therapies approved for the treatment of yellow fever. Supportive care includes maintenance of nutrition, prevention of hypoglycemia, treatment of hypotension with fluid replacement, administration of oxygen, prophylactic anticonvulsant therapy, management of metabolic acidosis, treatment of hemorrhage with fresh frozen plasma, and dialysis in cases of renal failure [11,12]. Antivirals such as ribavirin and sofosbuvir are under investigation, but clinical efficacy has not yet been proven [13,14]. Recent studies also suggest that MEK/ERK pathway inhibitors may have potential as antiviral therapies against YFV [15].

In Brazil, yellow fever vaccination is carried out through the administration of immunobiologicals derived from the 17D strain of the attenuated virus, widely used in campaigns by the National Immunization Program (PNI). The main vaccines employed are 17DD, produced by the Institute of Immunobiological Technology (Bio-Manguinhos/Fiocruz), and 17D-213, manufactured by the Institute of Viral Vaccine Production (Sanofi Pasteur). Both exhibit high immunogenicity and provide long-lasting protection against the disease. Since 2017, following the recommendations from the World Health Organization (WHO) and the Ministry of Health, the vaccination schedule adopted in the country consists of a single lifetime dose, with no need for a booster. This strategy ensures broad population coverage and contributes to the prevention of outbreaks in endemic and high-risk areas [11,13].

In addition to vaccination, additional prevention measures include avoiding mosquito bites by using repellents, protective clothing, and mosquito nets, especially in high-risk areas. Continuous epidemiological surveillance and implementation of vector control programs are crucial to reducing the YFV transmission [13,14]. 

Continued research is vital to develop more effective treatments and to better understand how the virus interacts with the host and environment. Recent studies have focused on identifying new antivirals, understanding the molecular biology of the virus, and the host immune response. For example, MEK/ERK pathway inhibitors and other antiviral compounds are being investigated for their potential to inhibit the YFV replication [15].

International collaboration and information sharing are essential to combat yellow fever as a global health threat. The World Health Organization (WHO) and other international entities play a crucial role in coordinating vaccination efforts, epidemiological surveillance and research. Cooperation between countries is essential to address yellow fever outbreaks, especially in regions where health infrastructure is limited.

## 2. Climate Change and Deforestation

Climate change and deforestation have a significant impact on the distribution of yellow fever-transmitting mosquitoes, such as *Aedes aegypti*. Rising global temperatures and changing precipitation patterns create favorable conditions for the proliferation of these vectors in previously non-endemic areas [16]. Spatial modeling using geographic information systems and *CLIMEX* suggests that climate change may expand the geographic habitable area for *Aedes aegypti*, increasing the risk of yellow fever outbreaks in urban areas [16].

Environmental change caused by deforestation also contributes to the spread of yellow fever. Forest destruction alters the natural habitats of mosquitoes and non-human primates, forcing these vectors to adapt to urban and peri-urban environments [17,18,19]. Furthermore, deforestation can increase human exposure to wild vectors, facilitating the transition of sylvatic yellow fever to urban areas [20].

Studies have shown that climate change directly influences the persistence and competence of *Aedes aegypti* and *Ae. albopictus* mosquitoes to transmit viruses, including the yellow fever virus. Rising global temperatures may extend the transmission season and increase the development rate of mosquitoes, resulting in a higher incidence of yellow fever [21,22]. Modeling suggests that with continued global warming, areas previously considered low-risk may become favorable for yellow fever transmission [16,22].

The combination of climate change and deforestation creates a scenario conducive to the expansion of yellow fever into new areas. The global distribution of *Aedes aegypti* is highly influenced by climatic factors, and regions suffering from deforestation are particularly vulnerable to outbreaks [18,19]. In some areas of Africa, for example, yellow fever transmission varies with the seasons, being most intense during the rainy season, when mosquito populations increase [18].

The seasonal distribution of yellow fever and the climatic conditions that favor the proliferation of vector mosquitoes emphasize the need for control strategies that consider environmental variables [18]. The adaptation of mosquitoes to new environmental conditions due to deforestation and climate change reinforces the need for continuous monitoring and an integrated public health response [18,19].

Yellow fever remains a significant threat in the tropical regions of sub-Saharan Africa and South America. It is transmitted primarily by the vector *Aedes aegypti* in urban areas and by *Haemagogus* and *Sabethes* mosquitoes in sylvatic cycles [23].

The disease presents ongoing challenges due to frequent outbreaks in densely populated areas with insufficient vaccination coverage. In 2013, an estimated 130,000 cases of viscerotropic disease and 78,000 deaths occurred in Africa, highlighting the severity of the health impact of yellow fever [24]. The 2016 epidemic in Angola and the Democratic Republic of the Congo, which resulted in the emergency distribution of 30 million vaccine doses, highlights the vulnerability of urban populations [25].

In Brazil, yellow fever has presented significant challenges due to its reemergence in areas that had been free of the disease for more than 70 years. Between 2016 and 2018, the country faced the largest outbreak in the last eight decades, with the southeast region being particularly affected [26]. The rapid expansion of the disease to large urban areas required a reassessment of vaccination and vector control strategies.

The continued circulation of the virus in the urban areas of Minas Gerais between 2021 and 2023 indicates a persistent risk of reurbanization of transmission, despite eradication efforts [27]. The situation is exacerbated by the widespread presence of the *Aedes aegypti* vector, capable of sustaining urban transmission cycles.

Globally, yellow fever remains a significant threat due to urban expansion and increased international travel. Introduction of the virus into non-endemic areas, such as Asia, through unvaccinated travelers could have devastating consequences due to the lack of specific immunity in local populations and the prevalence of the urban vector [11].

The epidemiological scenario of yellow fever underscores the need for continued surveillance, adaptive immunization strategies, and robust preparedness to respond to outbreaks. Mass vaccination campaigns and genomic monitoring are essential to prevent the spread of the disease and protect at-risk populations.

### Epidemiological Monitoring

YF not only exemplifies the challenges faced in managing vector-borne diseases, but also highlights the importance of epidemiological surveillance and public health emergency preparedness. By strengthening the local capacities for a rapid diagnosis, appropriate treatment, and implementation of robust preventive measures, we can hope to keep this disease under control and protect the vulnerable populations worldwide [3,11,13,14].

This study investigates the epidemiology of YF in Brazil from 2011 to 2020, discussing the impact of environmental changes, vaccination coverage and socioeconomic factors on the incidence and mortality of the disease, focusing on its trend and distribution in the territory.

## 3. Methods

This is an analytical, ecological time-series study carried out in the 27 federative units of Brazil. The country has a territorial area of approximately 8,510,000 km^2^, 5570 municipalities, and an estimated population of 203 million inhabitants. Regarding the population density, according to the last census in 2022, the country had 23.8 inhab./km^2^; in addition, it had a Human Development Index (HDI) equivalent to 0.760 [28]. 

The data studied were obtained through the Department of Information and Informatics of the Unified Health System (DATASUS) referring to confirmed cases of yellow fever that occurred between 2011 and 2020 [29].

Socioeconomic data were collected from digital sources at the Brazilian Institute of Geography and Statistics (IBGE), the Institute of Applied Economic Research (IPEA), and DATASUS. Epidemiological data on the disease were collected by DATASUS. For the time-series analysis, the data will be organized in spreadsheets using Excel software, over a 10-year period, and the average for the period will be extracted, followed by the incidence per 100,000 inhabitants. The sociodemographic data used in this research were: absolute cases, incidence, complications, and deaths. The health indicators were yellow fever (YF) vaccine coverage, percentage (%) of primary health care coverage (PHC), life expectancy, infant mortality, and fertility rate. The socioeconomic indicators were the illiteracy rate, GINI Index, Municipal Human Development Index (MHDI), percentage (%) of the Bolsa Family, and Gross Domestic Product (GDP) per capita [30].

## 4. Data Analysis Methodology

To analyze the trends, Prais–Winsten autoregressive models were used, in which the dependent variables were the annual incidence coefficients per hundred thousand inhabitants per regional health unit (URS), and the years of the study (2011 to 2020) were used as independent variables. The Prais–Winsten regression model was adopted because it is indicated to correct serial autocorrelation from time series.

To perform the Prais–Winsten regression, the annual incidence of AF was transformed both by year and by UF to the logarithmic scale. This process is performed to reduce the heterogeneity of the variance of the residuals resulting from the regression analysis of the time series.

The average annual percentage change (APC) was also calculated for each dependent variable analyzed. The following formula was used to calculate the APC:

APC = (−1 + 10[b1] ∗ 100%), where b1 refers to the angular coefficient (beta) of the Prais–Winsten regression (Antunes, 2015) [31].

The 95% confidence intervals (95%CI) of the APC measurements were also calculated using the following formula: minimum 95%CI (−1 + 10 [b1 – t ∗ e] ∗ 100%) and maximum 95%CI (−1 + 10 [b1 + t ∗ e] ∗ 100%). The values of the angular coefficients (b1) of the Prais–Winsten regression and standard errors were generated by a statistical analysis program. The t in the formula refers to Student’s *t*-test, which corresponded to 9 degrees of freedom (t = 2.262 for the ten-year period) with a 95% confidence level.

The regression results were interpreted as follows: increasing trend, when the *p*-value was less than 0.05 and the regression coefficient was positive; decreasing trend, when the *p*-value was less than 0.05 and the regression coefficient was negative; or stationary trend, when the *p*-value was greater than 0.05.

For the spatial analysis, cases were geolocated from the municipality of infection according to the absolute confirmed cases and subsequently identified risk areas according to the incidence of cases per hundred thousand inhabitants.

## 5. Bivariate Global and Local Spatial Autocorrelation

The bivariate global Moran’s I was calculated to verify the correlation between a variable in one municipality and a different variable in neighboring municipalities that belong to the entire study area.

This statistic provides an indication of the degree of linear association (positive or negative) between the value of a variable in one location and the mean of another variable in neighboring locations. The null hypothesis is spatial independence, in which case its value would be zero. The statistic can range from −1 to +1. Positive values (between 0 and +1) indicate direct correlation, and negative values (between 0 and −1) indicate inverse correlation [29,30].

Since the global Moran’s I provides a single value as a measure of spatial association for the entire data set, and does not demonstrate local patterns of spatial association, the bivariate local Moran’s I was calculated. In this technique, only the municipalities defined in a spatial neighborhood matrix are included in the calculation. This index provides the degree of statistically significant spatial autocorrelation in each unit. A greater similarity of the data than that of spatial randomness suggests a spatially similar grouping with the two variables.

A dissimilarity greater than that of spatial randomness would imply a strong, local, and negative relationship between the two variables. The local analysis is visualized in the form of Local Indicators of Spatial Association (LISA) cluster maps. For the bivariate local spatial correlation analysis, the Queen contiguity matrix of order one, calculated in the GeoDa^®^ software, version 1.12 (https://spatial.uchicago.edu/geoda, accessed on 26 February 2025), which considers regions that have common borders as neighbors, was used.

The pseudo *p*-value with 999 permutations was used to assess the significance of 1%, both for the global Moran’s I and for the local Moran’s I 14.

For data analysis, the Statistical Software for Professionals (Stata) statistical package, version 16.0, was used. Since these are non-nominal public data, available through the DATASUS database, they were not submitted for evaluation by the Research Ethics Committee.

## 6. Results

In Brazil, yellow fever cases are distributed in the following Amazon and Atlantic Forest biomes, predominantly due to their intrinsic environmental characteristics: the Amazon located in northwestern Brazil (in the states of Acre, Amapá, Amazonas, Pará and Roraima, and partially in Maranhão, Mato Grosso, Rondônia and Tocantins), and other countries in South America (Peru, Colombia, Venezuela and Guyana) and the Atlantic Forest located along the east coast of Brazil, from Rio Grande do Norte to Rio Grande do Sul (up to parts of Paraguay and Argentina).

**Figure 1:**
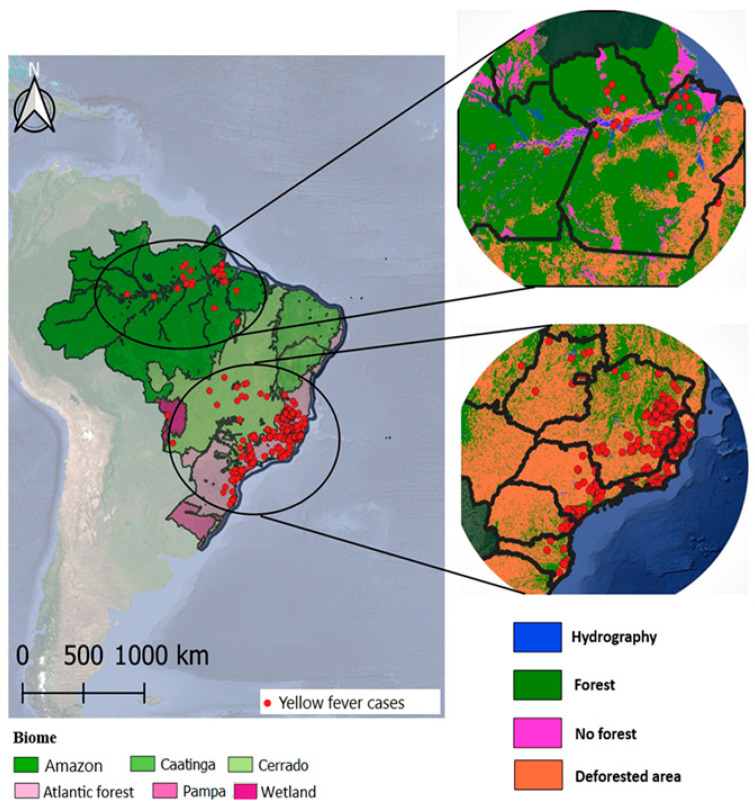
Yellow fever cases in Brazil according to biome and environmental characteristics. Source: SVS, 2024, PRODES, 2024. Prepared by Helierson Gomes in 2024.

The first has an equatorial climate and the second has a humid tropical climate, mainly sharing high temperatures and significant humidity throughout the year, which provides a soil rich in decomposing residues, a diverse fauna and its representatives, the non-human primates, dense forests interspersed with igapós and floodplains (in the Amazon), in addition to significant challenges due to the deforestation resulting from agricultural/urban expansion, mining, and burning. Thus, these climates constitute environments favorable to the proliferation of vectors of the genera Haemagogus, Sabethes and Aedes, and their contact with humans in the fragmentation of forests and the approximation of urban and rural areas, as evidenced in Figure 1.

The data in Table 1 on the epidemiology of yellow fever cases in the state of Pará (the only one with an increasing trend in the series (2011–2020)) indicate a predominance of cases in male individuals (83.3%), among black, brown, and yellow individuals (94.3%), with indigenous people in a distinct category due to 69,180 thousand people living on indigenous lands in this state (the 6th largest contingent in the country). The laboratory criterion was used in 70% of diagnoses and most cases were cured (96.3%), with only 3.7% of deaths.

In terms of occupation, farmers represent the majority of cases (28%), followed by rural workers (18%) and students (14%). Regarding education, most cases have up to 8 years of schooling (34%), followed by the group of up to 4 years, and in third place the group up to 12 years (16%), with zero representatives having more than 12 years of schooling. A total of 22% of the information on occupation and 14% on schooling was missing.

In the analysis of the trend of confirmed cases of yellow fever in the different states of Brazil in the years 2011–2020, between the absolute numbers and their percentage incidence, including the annual percentage for an estimated confidence interval of 95% for each region, there was mostly a stationary trend, with the exception of Pará and Santa Catarina with increasing trends in absolute cases, and only Pará in terms of percentage incidence (Table 2).

**Figure 2:**
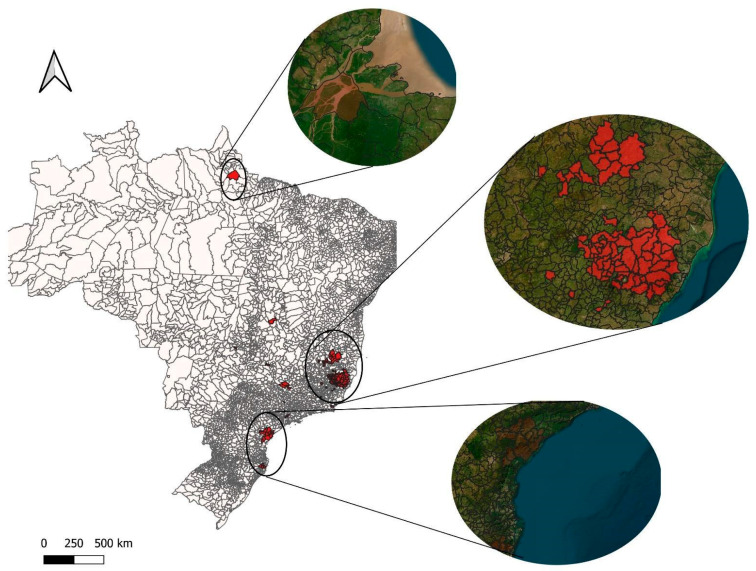
Areas at risk for yellow fever infection (incidence > 20 cases per 100,000 inhabitants).

Regarding the areas at risk for yellow fever infection (where an incidence of >20 cases per 100,000 inhabitants was considered), a predominance of the southeast region including the states of Minas Gerais, Espírito Santo, and São Paulo became evident; followed by the south region represented by Santa Catarina and Paraná; the state of Pará in the north region; Goiás in the center-west; and Maranhão in the northeast (Figure 2).

**Figure 3:**
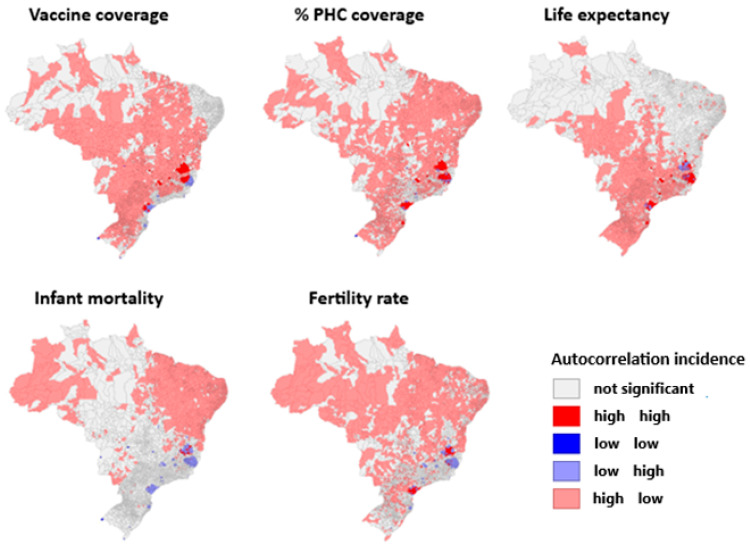
Spatial autocorrelation between health indicators and yellow fever incidence levels in Brazil.

In turn, Figure 3 illustrates the spatial autocorrelation between health indicators and yellow fever incidence levels in Brazil according to the Moran index. Municipalities with high vaccination coverage and low occurrence of yellow fever are the majority in the national territory, even in areas at risk for the pathogen, such as the Amazon biome, except in certain regions of endemic foci in the southeast (Minas Gerais and Espírito Santo) that have a high vaccination coverage and a high incidence of yellow fever, which are close to the regions with low vaccination occurrence and a high amount of cases of yellow fever, both representing a minority in the Brazilian panorama. There is no significance in the northeast and north regions.

Regarding primary health care coverage, most of the northern territory does not demonstrate significance; however, the northeast, followed by the south and central west demonstrate the lower incidence of yellow fever the greater the primary care coverage, while in the southeast the endemic foci also have an adequate primary health care network. The life expectancy parameter, for the south, central west, and southeast regions, is higher in conjunction with lower records of yellow fever. Records also show a high life expectancy and high incidence of the disease in the endemic foci in the southeast, with no significance in the northeast and further north.

In contrast to the predominance of infant mortality existing in the far west of the northern region and in the northeast region, which both show reduced occurrence of yellow fever, the high foci of the disease appears in Minas Gerais, Espírito Santo, and the south, which report low mortality rates in children up to 1 year of age per 1000 live births. The other locations lack proven significance and represent the largest national portion.

The tendency to automatically correlate the fertility rates with the high southeastern foci of the disease alternates between low fertility and high disease rates and vice versa, while in the south, the regions with a higher incidence of yellow fever also have high fertility rates. The north and northeast regions have a greater number of live births per woman throughout the fertile period, in contrast to the lower records of the endemic disease.

**Figure 4:**
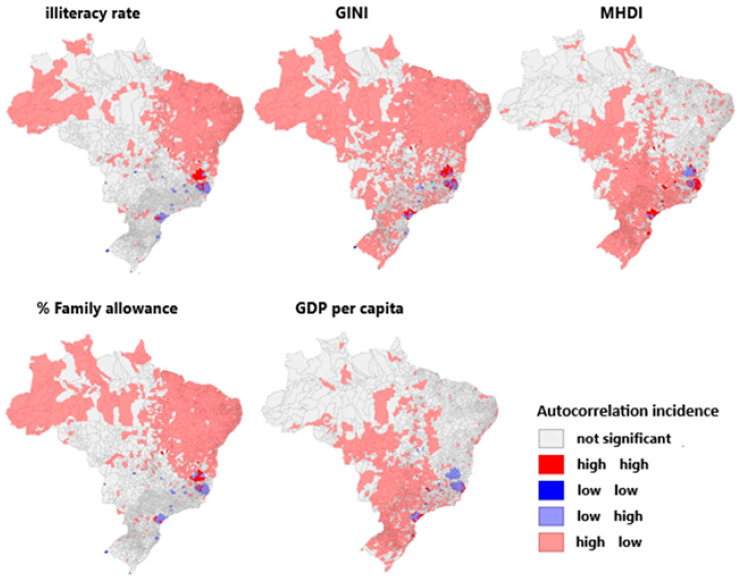
Spatial autocorrelation between socioeconomic indicators and yellow fever incidence levels in Brazil.

Regarding the socioeconomic indicators analyzed in Figure 4 and the spatial autocorrelation with the incidence of yellow fever in Brazil, the illiteracy rate does not present a significant correlation in most of the Brazilian territory. A significant correlation presents only in the north and northeast regions, where a high amount illiterate people correlates with a low incidence of yellow fever, and in the southeast region, where the opposite occurs. Regarding the income concentration rate (GINI), it is possible to identify a positive autocorrelation (high–high) in several municipalities located in the southeast region.

Regarding the municipal human development index (MHDI), there was no significance in the north and northeast, with a high MHDI in the other regions, presenting simultaneously with only a few records of the disease. This trend was reversed to the percentage of Bolsa Família, maintaining the pockets of divergence in the southeast endemic foci in both cases. A high incidence of yellow fever with a low MHDI and % of Bolsa Família, respectively, was interspersed with areas with opposite patterns. The GDP per capita, the average of the wealth produced divided by the number of inhabitants, followed the pattern of the MHDI, except for the risk foci in the southeast that presented low GDP per capita and high rates of yellow fever—unlike the other parameters in which these pockets have both high–high and low–high patterns.

**Figure 5:**
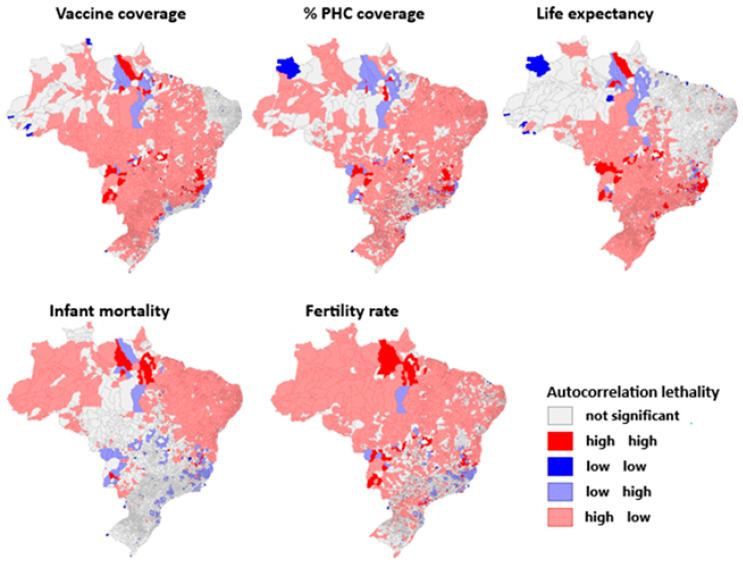
Spatial autocorrelation between health indicators and yellow fever mortality levels in Brazil.

Figure 5, which shows the spatial autocorrelation between health indicators and the levels of yellow fever lethality in Brazil, shows that high values of vaccination coverage correlate with few deaths from yellow fever in most of Brazil except for small areas in the southeast, extreme center west, and north (Amapá and Pará), where many deaths are recorded despite good vaccination coverage. In turn, a strip in the center of the northern region and on the southeast coast presents low vaccination coverage coinciding with many deaths, with small portions of the low-low pattern remaining in the extreme north. The pattern is maintained by the primary health care coverage parameter; however, there is an expansion in the extreme north, where a low-low pattern is present and on the northeastern coast, where a better health network coexists with few deaths from the disease (both being locations where the autocorrelation with vaccination coverage did not demonstrate significance).

Regarding the life expectancy, the locations with low incidence and simultaneous high death rates from yellow fever are present in the center north, while parts of Amapá, the southern part of the center west, and the southeast coast experience higher life expectancy associated with a higher mortality. There is no significance in the other northern locations and in the northeast.

Locations with low incidence of infant mortality and low fertility rates presenting simultaneously with high rates of death from yellow fever are present in the north central region and the southern part of the central west region. However, despite these areas being close to high rates of both of the parameters associated with high lethality of the disease, in terms of fertility, the high–high pattern covers a larger area. It covers a larger area also in relation to the increase in infant mortality and fertility rate coinciding with the low lethality of yellow fever—predominant in the national context in both cases, but higher in terms of fertility. An inverse pattern occurs in terms of the non-significance that predominates in the map of infant mortality.

**Figure 6:**
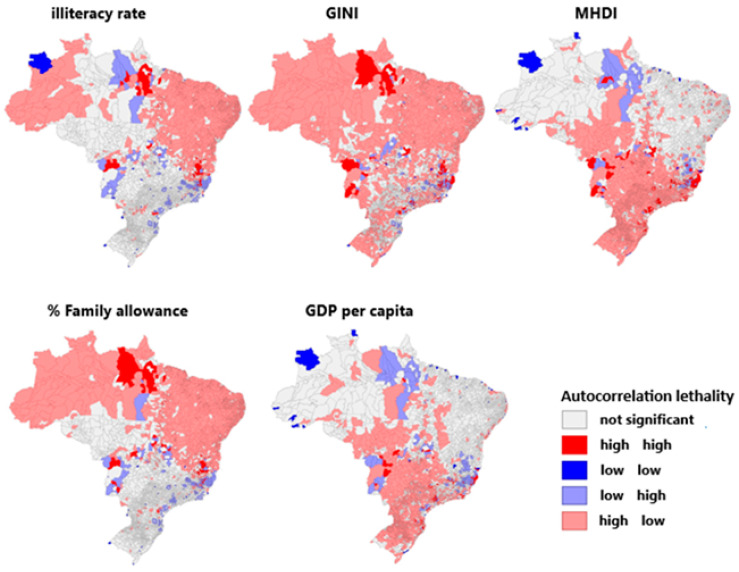
Spatial autocorrelation between socioeconomic indicators and yellow fever lethality levels in Brazil.

Regarding the spatial autocorrelation between the socioeconomic indicators and yellow fever lethality levels in Brazil (Figure 6), the illiteracy rate is predominantly high with low mortality in the extreme north and throughout the northeast, with the central north, the northeastern coast, and the southern part of the central west having a more educated population associated with higher mortality from yellow fever, with non-significance predominating in the other locations in Brazil (which represent the majority). However, there is a high income concentration rate coinciding with lower lethality at the national level, except for pockets of high concentration associated with high deaths in the central north and south of the central west.

There was no significance in the correlation of the HDI-M in most northern and northeastern municipalities, with a pattern of high HDI-M and low mortality from yellow fever in the other Brazilian regions, interspersed with high HDI-M and high deaths in the southeast coast and extreme north of the center west. There was a low development index in municipalities in the extreme north and northeast related to lower lethality rates of the disease and associated with high mortality in the center north and the southeast-northeast border.

In turn, the largest area in terms of percentage autocorrelation of Bolsa Família in Brazil is non-significant, with a distinction between the north and the northeast, with an increase in this percentage and a reduction in the records of deaths from yellow fever, with areas with a higher percentage of Bolsa Família associated with a higher mortality from the disease in the center north (regions in Amapá, Pará and Amazonas). The non-significance of autocorrelation also predominates in terms of GDP per capita, but this time represented by the north and northeast—except for the foci of a low–low pattern (similar to that evidenced in the maps of illiteracy rates and HDI-M), with records of low lethality and high GDP per capita in the south, southeast, and center west interspersed with the surrounding areas of low GDP per capita associated with high death rates in the center north, southeast coast, and southwest of the center west.

## 7. Discussion

An epidemiological analysis of yellow fever in Brazil has revealed dynamic patterns of incidence, influenced by environmental, socioeconomic, and public health determinants. Therefore, it is necessary to integrate the results of the geographic variations with those of isolated outbreaks in the control and prevention of yellow fever, considering the climatic variations that expand the risk zones.

The data analyzed previously, and other data, show that yellow fever remains predominantly sylvatic, with its epicenters located in the north and central west regions of Brazil, where the cycle of the *Haemagogus* and *Sabethes* vector predominates. However, the data indicated a much larger number of cases originating from the southern and southeastern states of the country, such as Santa Catarina and Minas Gerais, suggesting the geographic expansion of the disease to areas where it did not previously exist.

These factors, deforestation and climate change, set the conditions under which vectors spread to urban and peri-urban areas [16,18]. The analysis of incidence per 100,000 inhabitants showed that outbreaks were important in the most populous states, such as Minas Gerais and São Paulo, in 2017 and 2018, with cases in urban areas surrounding forests.

The recent yellow fever epidemic in Brazil has highlighted the challenges of controlling the disease in the context of environmental change. The international spread of the virus from the outbreak in Brazil illustrates the difficulty of containing infectious diseases in a highly mobile world. Infected travelers have carried the virus to other regions, including Asia, where yellow fever was not previously found [20,25]. This demonstrates the urgent need to strengthen epidemiological surveillance and vaccination campaigns in response to climate change and deforestation.

The spread to urban areas is a major concern for public health authorities, as it has risks the reintroduction of the urban cycle of yellow fever involving *Aedes aegypti*, as seen in the early 20th century [23]. Although the urban cycle has not yet been demonstrated, uncontrolled urbanization and population growth in previously isolated areas represent an imminent risk.

Yellow fever still infects mainly men of working age, implying an occupational exposure during rural and forestry work. Most cases were still concentrated in farmers, agricultural workers, and security guards. This reinforced the previous findings, where occupational vulnerability to exposure to the virus was a major determinant [3].

The absolute number of cases among non-white individuals and those with little education would confirm that social inequality leads to greater vulnerability to the disease and suggests that it is especially the economically disadvantaged groups that are predisposed to exposure, as their economic situation does not allow them to obtain adequate health care and preventive services [32].

Another relevant point is the distribution of cases by age, as they are more prevalent in young and middle-aged adults, in direct relation to the nature of occupational activities in the risk areas. The lack of informational campaigns aimed at these groups highlights the need for specific programs to increase vaccination adherence and the adoption of personal protection measures among rural workers and populations living in areas close to forests [33]. 

Vaccination is certainly the main measure to prevent yellow fever, but the results show that it is not distributed homogeneously throughout Brazil. States such as São Paulo and Minas Gerais carried out vaccination campaigns after the 2017 outbreak, but had lower vaccination coverage than the target ensuring herd immunity, especially in rural and peri-urban communities [25].

In 2017, the use of fractional doses of vaccines was an emergency tool to expand vaccination coverage in a short period of time, especially in high-risk areas. However, the long-term impacts of such a strategy remain largely unaddressed, particularly on the duration of immunity provided by fractional doses. It would be interesting to compare the immunity conferred by full and fractional doses, as well as possible vaccine boosters, in establishing protection.

Another key issue to consider is the logistics of vaccine distribution. The challenges in the logistics of vaccination campaigns make it difficult to implement vaccination activities in remote and hard-to-reach areas, leaving populations at risk for potential outbreaks. In addition, the underreporting of cases from these regions is also a problem, which makes it difficult to properly assess the true vaccination coverage and yellow fever incidence.

Directly related to climate change, in addition to the impact of deforestation, is the spread of vectors to new areas, mainly in southern Brazil. The increase in temperature and the altered distribution of rainfall are cited as factors that would have favored the proliferation of the mosquito in areas previously considered low-risk [21]. The expansion of the sylvatic cycle of yellow fever into peri-urban areas has been driven by uncontrolled urbanization and deforestation, processes primarily resulting from irregular land occupation. This scenario, particularly evident in the north and central west regions of Brazil, intensifies the interaction between human populations and disease vectors, increasing the risk of transmission and enhancing the potential for outbreaks in the previously non-endemic areas [34,35].

All of these factors point to the need for the continuous monitoring of the yellow fever transmission cycle in light of climate change. Implementing environmental surveillance programs to monitor mosquito populations and identify the presence of the virus in vectors and non-human primate populations at the borders of their distribution area would significantly contribute to health surveillance in these areas—an essential scenario for anticipating outbreaks and responding more quickly to new foci of infection.

In fact, yellow fever remains highly lethal, particularly among the severe cases with complications such as liver failure, necrotizing pancreatitis, and kidney failure. Data show that mortality is higher in the areas with less access to health services, especially in the rural and remote areas. This suggests that, in addition to vaccination, strengthening health infrastructure in low-access areas is critical in the fight against mortality associated with the disease.

Age and the presence of underlying chronic diseases, especially diabetes and hypertension, as well as the associated comorbidities are considered prognostic factors for mortality during the clinical care of severe cases. Standardized protocols for the management of these cases in an intensive care setting could lead to better clinical outcomes, particularly in low-resource settings.

The main limiting factor in the present study is that the data were obtained from secondary sources, particularly DATASUS, which poses the risk of underreporting, especially in rural and remote areas. In addition, the lack of detailed information on population immunity and the consequences of fractional vaccinations limit the scope of a long-term evaluation of the interventions performed. Future studies should consider more rigorous data collection measures, focusing on the quality of epidemiological surveillance.

## 8. Conclusions

The results from this study support the need for a multidisciplinary approach to control yellow fever in Brazil, involving broader and more efficient vaccination campaigns, preferably in a timely manner, in addition to intensified vector control and active epidemiological surveillance. Vector control in urban environments that combines efforts to eliminate *Aedes aegypti* breeding sites is crucial to preventing the reurbanization of yellow fever, particularly in areas where human population density is growing rapidly. In this way, the predictive capacity of modeling helps to detect the areas at risk and allows for preventive interventions before outbreaks occur. This will require the coordination of public health agencies, environmentalists, and researchers, working in unison to respond to the types of risks that a rapidly changing environment creates.

Yellow fever remains a public health issue for Brazil due to the geographic expansion of risk areas and the influence of environmental changes. The geographic expansion of the disease and the increased incidence in urban and peri-urban areas show that this has not been an easy challenge. Future research should aim to develop new, more effective vaccines, monitor population immunity, and develop rapid responses to new outbreaks, including via partnerships between the health, environmental, and government sectors to ensure the safety of all vulnerable populations.

In summary, yellow fever is a complex disease that requires a multidisciplinary approach for its control and prevention. From the detailed virology of the YFV to the host immune response and effective prevention strategies, every aspect of the disease needs to be well understood to address the challenges it presents. Strengthened international collaboration, continued surveillance, and innovative research are key to protecting public health globally, and eventually eradicating yellow fever as a significant threat.

## Figures and Tables

**Table 1:** Epidemiological characteristics of yellow fever cases in the State of Pará, Brazil, 2010 to 2021.

Sex	*n*	%
Feminine	386	16.7
Masculine	1925	83.3
Race/Color		
White	40	1.7
Not white	2179	94.3
Indigenous	92	4
Confirmation criteria		
Laboratory tests	1617	70
Epidemiological data	694	30
Case Evolution		
Cure	2226	96.3
Death by AF	85	3.7
Occupation		
Farmer	647	28
Vigilant	47	2
Rural worker	416	18
Mason	139	6
Student	323	14
From home	231	10
Ignored	508	22
Education		
Illiterate	138	6
Up to 4 years	693	30
Up to 8 years	785	34
Up to 12 years old	369	16
>12 years	0	0
Ignored	323	14

**Table 2:** Yellow fever trend in states with confirmed cases in Brazil.

Health Region	2011	2012	2013	2014	2015	2016	2017	2018	2019	2020	APC	95% CI	Trend
Absolute Cases (n)											%		
Amazonas	0	0	2	0	0	2	1	1	1	0	0.04	−0.12; 0.21	Stationary
Para	2	0	1	1	2	0	8	0	4	3	0.36	0.06; 0.66	Growing
Tocantins	0	0	0	0	0	0	1	0	0	0	0.01	−0.05; 0.09	Stationary
Federal District	0	0	0	0	0	0	2	0	0	0	0.03	−0.10; 0.18	Stationary
Goias	0	0	0	0	6	3	1	0	0	0	1.54	−0.54; 0.54	Stationary
Mato Grosso	0	0	0	0	0	0	1	0	0	0	0.19	−0.05; 0.09	Stationary
Mato Grosso do Sul	0	0	0	0	1	0	0	0	0	0	0.17	−0.04; 0.08	Stationary
Minas Gerais	0	0	0	0	0	48	485	0	0	0	8.58	−25.4; 42.5	Stationary
São Paulo	0	0	0	0	0	2	86	505	74	0	20.7	−15.9; 57.4	Stationary
Espirito santo	0	0	0	0	0	0	253	3	0	0	5.1	−13.5; 23.8	Stationary
Rio de Janeiro	0	0	0	0	0	0	28	279	0	0	9.9	−9.3; 29.2	Stationary
Santa Catarina	0	0	0	0	0	0	0	0	2	20	1.14	0.01; 2.27	Growing
Incidence %	2011	2012	2013	2014	2015	2016	2017	2018	2019	2020	APC	95% CI	Trend
Amazonas	0	0	0.05	0.00	0.00	0.05	0.03	0.03	0.03	0	0.001	−0.000; 0.003	Stationary
Para	0.02	0.00	0.01	0.01	0.02	0.00	0.10	0.00	0.05	0.04	0.001	0.001; 0.008	Growing
Tocantins	0	0	0	0	0	0	0.06	0	0	0	0.001	−0.003; 0.005	Stationary
Federal District	0	0	0	0	0	0	0.07	0	0	0	0.001	−0.003; 0.006	Stationary
Goias	0	0	0	0	0.09	0.04	0.01	0	0	0	−0.000	−0.009; 0.009	Stationary
Mato Grosso	0	0	0	0	0	0	0.03	0	0	0	0.000	−0.001; 0.002	Stationary
Mato Grosso do Sul	0	0	0	0	0.03	0	0	0	0	0	0.000	−0.002; 0.002	Stationary
Minas Gerais	0.00	0.00	0.00	0.00	0.00	0.23	2.36	0.00	0.00	0.00	0.04	−0.13; 0.23	Stationary
São Paulo	0.00	0.00	0.00	0.00	0.00	0.00	0.19	1.14	0.17	0.00	0.04	−0.03; 0.13	Stationary
Espirito Santo	0	0	0	0	0	0	6.60	0.08	0	0	0.13	−0.35; 0.62	Stationary
Rio de Janeiro	0	0	0	0	0	0	0.17	1.74	0	0	0.06	−0.05; 0.18	Stationary
Santa Catarina	0	0	0	0	0	0	0	0	0.03	0.26	0.01	−0.004; 0.04	Stationary

## Data Availability

The data presented in this study are available upon request 435 from the corresponding author.
